# Validation of the Korean version of the Clinical Opiate Withdrawal Scale

**DOI:** 10.3389/fpubh.2025.1706053

**Published:** 2026-01-30

**Authors:** Jiseon Jang, Taeyoung Lee, Aram Im, Myungji Ha, Jinyoung Oh, Ki Beom Park, Dong Hyuck Kim, Hyo Shin Kang

**Affiliations:** 1 Department of Neuropsychiatry, Seoul National University Hospital, Seoul, Republic of Korea; 2 Department of Psychiatry, Kyungpook National University School of Medicine, Daegu, Republic of Korea; 3 Department of Psychiatry, Kyungpook National University Hospital, Daegu, Republic of Korea; 4 Bio-Medical Research Institute, Kyungpook National University Hospital, Daegu, Republic of Korea; 5 Department of Anesthesiology and Pain Medicine, Kyungpook National University School of Medicine, Daegu, Republic of Korea; 6 Department of Anesthesiology and Pain Medicine, Kyunpook National University Chilgok Hospital, Daegu, Republic of Korea; 7 Department of Anesthesiology and Pain Medicine, Keimyung University Dongsan Hospital, Keimyung University School of Medicine, Daegu, Republic of Korea; 8 Department of Anesthesiology and Pain Medicine, Daegu Catholic University School of Medicine, Daegu, Republic of Korea; 9 Department of Anesthesiology and Pain Medicine, Daegu Catholic University Medical Center, Daegu, Republic of Korea; 10 Department of Psychology, Kyungpook National University, Daegu, Republic of Korea

**Keywords:** K-COWS, opioid use disorder, opioid withdrawal, the Clinical Opiate Withdrawal Scale, validation

## Abstract

**Background:**

The use of prescription opioids has risen dramatically in South Korea in recent years; however, there is no standardized Korean-language instrument to assess opioid withdrawal. The Clinical Opiate Withdrawal Scale (COWS) is a widely used clinician-rated measure for assessing the severity of opioid withdrawal. We aimed to validate the Korean version of the COWS (K-COWS) among patients receiving opioid therapy.

**Methods:**

We translated and culturally adapted the 11-item COWS into Korean. A total of 66 adult patients with opioid use disorder who were experiencing withdrawal symptoms were assessed. Each patient was evaluated using the K-COWS and completed the Subjective Opiate Withdrawal Scale (SOWS), the brief 3-item Opioid Craving Scale (OCS-3), and the single-item Opioid Craving Visual Analog Scale (OC-VAS). We examined the internal consistency, factor structure [through exploratory factor analysis (EFA) and confirmatory factor analysis (CFA)], and convergent/discriminant validity of the K-COWS.

**Results:**

The K-COWS showed good internal consistency (Cronbach’s *α* = 0.79; 95% CI = 0.71–0.86). EFA supported a two-factor structure (physical vs. autonomic clusters) that explained 39% of the variance. In CFA, the two-factor model showed a marginally acceptable fit (*χ^2^* = 47.76, CFI = 0.915, RMSEA = 0.082), whereas the unidimensional alternative showed a poor fit. The K-COWS strongly correlated with the SOWS (*r* = 0.74, 95% CI = 0.61–0.83, *p* < 0.001), indicating good convergent validity for withdrawal. In contrast, correlations with the craving measures were weak (OCS-3: *r* = 0.19, *p* > 0.05; OC-VAS: *r* = 0.20, *p* > 0.05), supporting discriminant validity.

**Conclusion:**

The K-COWS is reliable and has demonstrated construct validity for withdrawal severity in Korean clinical settings. Given the preliminary nature of the factor structure, the total score is recommended for clinical decision-making. Future studies should assess inter-rater reliability and confirm the structure in larger, more diverse samples.

## Introduction

Opioid use disorder (OUD) is a chronic, relapsing medical condition characterized by compulsive use, impaired control, intense craving, and continued use despite negative consequences. This disorder is also marked by physiological adaptations such as tolerance and withdrawal (1). Prolonged μ-opioid receptor stimulation drives neuroadaptations in circuits related to reward, stress, and executive control; noradrenergic hyperactivity in the locus coeruleus contributes to the autonomic and somatic features of opioid withdrawal, which is a major driver of relapse and presents a major obstacle to recovery (2, 3). On a population level, OUD has a considerable and growing impact on morbidity and mortality, highlighting the urgency of enhanced clinical and public health responses (4). Contemporary neurobiological models of addiction provide a mechanistic framework for understanding the clinical phenomena observed during the binge/intoxication, withdrawal/negative affect, and preoccupation/craving stages (5). This underscores the need for standardized clinical assessments that can translate emerging biological insights into consistent clinical practice (6).

South Korea has seen a marked increase in medical opioid exposure in recent years. Nationwide analyses show that annual opioid prescription rates increased from 347.5 per 1,000 persons in 2009 to 531.3 per 1,000 in 2019, with the most notable growth observed in strong opioids, extended-release and long-acting formulations, and among older adults in tertiary care settings (7, 8). Although South Korea’s Narcotics Information Management System (NIMS) and health insurance claims database (HIRA) offer extensive surveillance of opioid dispensing and prescribing patterns, these administrative data sources are not designed to identify clinically verified OUD or to quantify withdrawal burden at the bedside (9). Thus, true OUD prevalence and clinical severity are difficult to estimate, particularly in non-psychiatric settings where long-term opioids are managed (e.g., chronic pain clinics). Patients with chronic pain may be reluctant to seek addiction treatment due to stigma, and OUD can go unrecognized without a structured assessment (10). Overall, Korea faces growing opioid exposure and early signs of misuse; however, the absence of standardized clinical measures, particularly for withdrawal, limits safer clinical management and effective surveillance.

Addressing opioid-related problems effectively relies on tools for risk stratification, diagnosis, and withdrawal assessment. For risk evaluation, the Opioid Risk Tool (ORT) and the revised Screener and Opioid Assessment for Patients with Pain (SOAPP-R) help estimate the likelihood of aberrant medication-related behaviors before or during chronic therapy, and the Current Opioid Misuse Measure (COMM) detects current misuse among patients already receiving opioids (11–13). Diagnosis follows the DSM-5 criteria, which distinguish physiological dependence from addiction severity. For assessing withdrawal, both patient-and clinician-rated scales exist. The Subjective Opiate Withdrawal Scale (SOWS) captures self-reported symptoms (14), whereas older clinician tools such as the Clinical Institute Narcotic Assessment (CINA) have provided an initial objective framework. The Clinical Opiate Withdrawal Scale (COWS) is the most widely used clinician-rated instrument to quantify withdrawal severity. It is brief and reproducible and has demonstrated acceptable internal consistency and concurrent validity compared to CINA and single-item ratings (15, 16). In practice, COWS thresholds are useful for determining the timing of buprenorphine initiation (typically when a patient is experiencing at least mild to moderate withdrawal, often indicated by a COWS score of 8–12). These thresholds also help track patients’ response to symptomatic or agonist treatment and standardize decisions regarding tapering. Cross-lingual adaptations (such as those in Turkish and French-Canadian) have demonstrated that the instrument is portable and has broadly similar psychometrics across settings (17, 18). Despite the rising clinical need, there has been no validated Korean clinician-rated withdrawal scale (K-COWS) to standardize assessments in Korean hospitals and clinics and to align Korean data with international literature.

Accordingly, we undertook the translation, cultural adaptation, and psychometric validation of the K-COWS, following established guidelines for cross-cultural instrument adaptation (19). Our objectives were threefold: (i) to evaluate internal consistency; (ii) to examine the factor structure; and (iii) to establish construct validity through convergent validity with the SOWS and discriminant validity against craving measures. By providing a brief, clinician-administered, Korean-language withdrawal measure that aligns with international best practices, this study aims to support routine clinical decisions and enable prospective monitoring and research on opioid-related outcomes in Korea.

## Methods

### Study design and participants

This multicenter, cross-sectional validation study was conducted at three university-affiliated hospitals in Daegu, South Korea: Kyungpook National University Hospital, Daegu Catholic University Medical Center, and Keimyung University Dongsan Medical Center. Participants were long-term prescription opioid recipients in pain clinics who met DSM-5 criteria for OUD and were assessed during a withdrawal episode. Eligibility required (i) current or recent long-term opioid therapy for pain and (ii) a DSM-5 diagnosis of OUD established via clinical interview by a board-certified psychiatrist. Participants were assessed during a clinically verified episode of opioid withdrawal (spontaneous or precipitated); a subset was evaluated during inpatient detoxification following referral from the pain clinics. Exclusion criteria included acute medical or psychiatric instability that could confound ratings, concurrent withdrawal from sedative-hypnotics, or medications likely to mask or mimic opioid withdrawal. All participants provided written informed consent. The study was approved by the Institutional Review Board of Kyungpook National University Hospital and conducted in accordance with the Declaration of Helsinki (no. 2025-02-005).

### Instruments and procedure

To adapt the COWS into Korean, we followed established guidelines for cross-cultural adaptation of measurement scales. First, two bilingual translators independently forward-translated the 11-item COWS from English to Korean, and the research team reviewed the draft for conceptual equivalence. Next, two independent translators performed back-translation, which was reconciled with the source text, and minor wording adjustments were implemented. An expert panel (one addiction psychiatrist, one pain specialist) was formed to evaluate content, cultural relevance, and face validity and agreed that the Korean version retained the meaning of the original. A small pilot test (*n* = 2) confirmed its comprehensibility with no further changes. The final K-COWS contains the same 11 items as the original COWS, each rated 0–4 or 0–5, yielding a total score range of 0–48; higher scores indicate more severe withdrawal (5–12 mild, 13–24 moderate, 25–36 moderately severe, ≥37 severe) (16). After enrollment, each participant underwent a structured assessment of withdrawal severity using the K-COWS, administered by a clinician trained in the COWS. In the same session, to assess convergent validity for the intended construct, participants completed the SOWS (16 items, 0–4 each; total 0–64) (14). The SOWS is a self-report questionnaire listing common opioid withdrawal symptoms (such as anxiety, nausea, and muscle aches), which participants rate based on how they currently feel; we used the 16-item version (each item rated 0–4; total score range 0–64). To evaluate discriminant validity with respect to craving (a related but distinct construct), participants completed two measures: the 3-item Opioid Craving Scale (OCS-3; each item rated 0–10; total score 0–30) (20) and the Opioid Craving Visual Analog Scale (OC-VAS), a single-item 0–10 VAS for current opioid craving (0 = no craving at all, 10 = strongest imaginable) (21).

### Statistical analysis

We evaluated the psychometric properties of the K-COWS using established procedures. Descriptive statistics summarized demographic and clinical characteristics and score distributions; between-sex differences in K-COWS, SOWS, OCS-3, and OC-VAS scores were examined with independent-samples t-tests. To assess reliability, the internal consistency of the 11 K-COWS items was evaluated using Cronbach’s alpha with 95% confidence intervals. We also examined corrected item–total correlations and alpha-if-item-deleted values. For construct validity, we conducted an exploratory factor analysis (EFA) using maximum likelihood extraction with oblique (oblimin) rotation after confirming sampling adequacy with the Kaiser–Meyer–Olkin (KMO) measure and Bartlett’s test of sphericity. The number of factors was determined primarily by Horn’s parallel analysis, with the scree plot and the Kaiser criterion (eigenvalues > 1) used as supportive evidence. Factor loadings < 0.30 were suppressed for presentation. Based on the EFA solution, we then fit a confirmatory factor analysis (CFA) and evaluated model fit using CFI, TLI, RMSEA, and SRMR. We compared the fit of the two-factor model derived from EFA with that of a unidimensional alternative. Conventional criteria to evaluate model fit based on CFI, TLI (acceptable fit ≥0.90, good fit ≥0.95), RMSEA, and SRMR (acceptable fit ≤0.08, good fit ≤0.05) were applied in this study (22, 23). Composite reliability (CR) and average variance extracted (AVE) were calculated from standardized loadings. Convergent and discriminant validity were examined using Pearson’s correlations between K-COWS and SOWS (convergent) and between K-COWS and OCS-3/OC-VAS (discriminant). All analyses were performed using R version 4.5.1. A two-tailed *p* < 0.05 was considered statistically significant.

## Results

### Participant characteristics

The sample consisted of 66 participants (42 males, 63.6%) with a mean age of 58.8 years (SD = 15.1). Table 1 summarizes their key demographic and clinical characteristics. No significant sex differences were observed in K-COWS, SOWS, OCS-3, or OC-VAS scores. Overall, withdrawal severity was in the mild-to-moderate range across all measures.

**Table 1:** Demographic and clinical characteristics (*N* = 66).

Variable	Total sample	Males (*n* = 42)	Females (*n* = 24)	*t*-value	*p*-value
Age, years	58.8 (15.1)	57.4 (14.8)	61.1 (15.6)	−0.96	0.341
K-COWS	8.7 (6.6)	8.31 (6.5)	9.46 (6.9)	−0.67	0.509
SOWS	19.7 (14.2)	20.14 (14.7)	18.83 (13.5)	0.37	0.715
OCS-3	14.5 (9.7)	15.50 (9.5)	12.83 (10.1)	1.05	0.297
OC-VAS	4.8 (2.9)	5.02 (2.9)	4.42 (3.0)	0.89	0.378

Values are mean (SD). K-COWS, Korean version of Clinical Opiate Withdrawal Scale; SOWS, Subjective Opiate Withdrawal Scale; OCS-3, Opioid Craving Scale; OC-VAS, Opioid Craving Visual Analog Scale.

### Reliability

The K-COWS demonstrated good internal consistency (Cronbach’s *α* = 0.79, 95% CI = 0.69–0.85), comparable to the original English version. Item-total correlations ranged from 0.27 to 0.68. While items 6 (runny nose or tearing) and 7 (GI upset) showed slightly weaker correlations (*r* = 0.27 and 0.32, respectively), no item showed a problematic correlation (<0.20) that would warrant removal. Other measures also showed excellent reliability (SOWS Cronbach’s α = 0.93; OCS-3 Cronbach’s *α* = 0.83).

### Construct validity

The Kaiser-Meyer-Olkin (KMO) measure (0.74) and Bartlett’s test of sphericity (*χ^2^* = 233.99, *p* < 0.001) confirmed the suitability of the data for factor analysis. Parallel analysis suggested a two-factor solution. The EFA identified a two-factor structure that accounted for 39% of the total variance (Table 2). Factor 1, which explained 20.6% of the variance, comprised physical withdrawal symptoms (items 2, 3, 8, 9, 10, and 11), while Factor 2 explained 18.4% of the variance and included autonomic symptoms (items 1, 4, 6, 7, and 11). Item 11 (gooseflesh skin) loaded on both factors, whereas Item 5 (bone/joint aches) showed poor loading on either. The factors demonstrated a moderate inter-factor correlation (*r* = 0.34, *p* < 0.01). Composite reliability was acceptable for both factors (Factor 1: CR = 0.77; Factor 2: CR = 0.73), although the average variance extracted was below the recommended threshold (AVE = 0.36 for both). The CFA yielded the following fit indices for the two-factor model: χ^2^ = 47.76 (*p* < 0.047); CFI = 0.915; TLI = 0.884; RMSEA = 0.082; SRMR = 0.083. Conversely, the unidimensional alternative model failed to meet acceptable fit thresholds (χ^2^ = 112.63; CFI = 0.650; TLI = 0.563; RMSEA = 0.153; SRMR = 0.125), supporting the scale’s multidimensional nature in this population.

**Table 2:** Factor structure of the K-COWS.

Item	Factor1 (Physical)	Factor 2 (Autonomic)	*h* ^2^
1. Resting pulse rate	—	0.50	0.26
2. Sweating	0.47	0.31	0.41
3. Restlessness	**0.81**	—	0.64
4. Pupil size	—	**0.55**	0.40
5. Bone or joint aches	—	—	0.17
6. Runny nose or tearing	—	**0.58**	0.35
7. GI upset	—	**0.80**	0.59
8. Tremor	**0.54**	—	0.28
9. Yawning	0.49	—	0.28
10. Anxiety or irritability	**0.72**	—	0.47
11. Gooseflesh skin	**0.51**	**0.52**	0.71
Variance explained (%)	20.6	18.4	
Composite reliability	0.77	0.73	
AVE	0.36	0.36	

K-COWS, Korean version of Clinical Opiate Withdrawal Scale; maximum likelihood extraction with oblimin rotation. Loadings < 0.30 suppressed. Bold indicates > 0.50. *h*^2^ = communality; AVE, average variance extracted. Inter-factor correlation = 0.34.

### Criterion-related validity

The K-COWS demonstrated strong convergent validity with the SOWS (*r* = 0.75, 95% CI = 0.61–0.83, *p* < 0.001), indicating good correspondence between clinician-rated and self-reported withdrawal symptoms. Discriminant validity was supported by the weak, non-significant correlations with the OCS-3 (*r* = 0.19, 95% CI = −0.05 to 0.41, *p* = 0.126) and with the OC-VAS (*r* = 0.20, 95% CI = −0.04 to 0.42, *p* > 0.05), confirming that the K-COWS specifically measures withdrawal rather than craving. The moderate correlation between the SOWS and OCS-3 (*r* = 0.37, *p* = 0.003) further supported the distinction between these constructs (Table 3).

**Table 3:** Correlations between K-COWS, SOWS, OCS-3, and OC-VAS (*N* = 66).

Scale	1	2	3	Cronbach’s α
1. K-COWS	-			0.801
2. SOWS	0.754^***^	-		0.931
3. OCS-3	0.201	0.366^**^	-	0.833
4. OC-VAS	0.231	0.351^**^	0.648^**^	-^a^

K-COWS, Korean version of Clinical Opiate Withdrawal Scale; SOWS, Subjective Opiate Withdrawal Scale; OCS-3, Opioid Craving Scale-3 items; OC-VAS, Opioid Craving Visual Analog Scale; ^**^p < 0.01; ^***^p < 0.001; ^a^Single-Item Scale, internal consistency not applicable.

## Discussion

This study established that the Korean version of the Clinical Opiate Withdrawal Scale (K-COWS) is a reliable and valid clinician-rated tool for assessing opioid withdrawal. In our psychometric evaluation, the K-COWS showed strong internal consistency, a plausible two-factor symptom structure, and the expected patterns of convergent validity (a high correlation with patient-reported withdrawal symptoms) and discriminant validity (a minimal correlation with craving measures). Clinically, these findings fill a critical gap: K-COWS now enables standardized withdrawal assessment in Korean medical settings for the first time. This study is particularly timely given the rising opioid use in Korea and the growing need for the standardized monitoring of withdrawal symptoms. By providing a Korean-language withdrawal instrument aligned with international standards, our study can improve bedside management of withdrawal and facilitate quantitative comparisons of OUD-related outcomes between Korea and other countries.

The K-COWS demonstrated good internal consistency, with a Cronbach’s alpha of 0.79 in our sample. This reliability is comparable to that of the original English COWS (16) and falls within the range of Turkish adaptations (17). Such consistency indicates that the translated items collectively measure the same underlying construct of withdrawal severity. Although we did not formally assess inter-rater reliability, prior studies have reported excellent inter-rater agreement for COWS (17). With standardized training and clear scoring guidelines, K-COWS is likewise expected to yield high agreement among clinicians. Overall, the internal consistency and expected inter-rater reliability support K-COWS as a dependable measure of opioid withdrawal symptoms.

Regarding construct validity, our EFA suggested two correlated latent dimensions—broadly “physical” vs. “autonomic” signs—that together explained 39% of the variance. CFA supported this structure, as the two-factor model showed significantly better fit indices than the unidimensional alternative. This pattern replicates the conclusion of Barbosa-Leiker et al. (24) that a single-factor structure does not adequately capture the covariance pattern among COWS items in complex clinical samples. Notably, the composition of our second factor, which includes ‘pulse rate,’ ‘pupil size,’ ‘runny nose or tearing,’ and ‘gastrointestinal upset,’ differs from the limited one- or two-item secondary factors that have been previously documented (17). This constellation reflects a coherent cluster of autonomic signs that are frequently emphasized in clinical descriptions of opioid withdrawal. The emergence of a more structured physiological factor extends earlier findings, suggesting that multidimensionality may be more interpretable in certain clinical populations. However, although the two-factor model fit was acceptable, some indices (e.g., TLI = 0.884) were marginal. This may be attributable to the specific characteristics of our sample—long-term prescription opioid patients treated in pain clinics—where symptoms such as ‘bone or joint aches’ may reflect underlying pain conditions or opioid-induced hyperalgesia rather than acute withdrawal itself (25, 26). Such symptom overlap introduces construct-irrelevant variance, meaning that pain-related items are less likely to fluctuate strictly in parallel with other withdrawal signs, thereby reducing factor loadings. Taken together, the cross-study pattern suggests that the COWS may not be strictly unidimensional across populations and that the interpretability of secondary factors depends on sample characteristics (e.g., clinical setting, opioid type). Given the preliminary nature of the dimensional structure and the noted limitations, the two-factor structure should be interpreted with caution. At present, the K-COWS can be applied most reliably as a total score for clinical decision-making, pending further refinement and replication of potential subscales in larger, more diverse samples.

K-COWS showed strong convergent validity, demonstrating high correlations with the Subjective Opiate Withdrawal Scale (SOWS), as clinician-observed signs increased in parallel with patients’ self-reported symptoms. This is consistent with previous findings from the original COWS validation (15). Equally important, K-COWS displayed discriminant validity by remaining largely independent of craving measures, underscoring that it measures withdrawal rather than generalized distress or urge to use. This separation is expected from neurocircuit models in which the withdrawal/negative affect stage and the preoccupation/anticipation (craving) stage are dissociable (5, 27, 28). Clinically, the distinction has consequences: craving can be high in the absence of substantial somatic withdrawal or vice versa, so clinicians should assess craving in parallel with withdrawal. Notably, higher OC-VAS scores predict subsequent opioid use during treatment, highlighting their complementary value for prognosis (21). Conversely, withdrawal severity indices inform acute management decisions, including the timing of agonist induction and symptomatic therapy (3). Thus, K-COWS isolates the physical withdrawal syndrome, while craving measures capture motivational risk; using both provides a fuller clinical picture and may improve safety and outcomes.

We observed no significant sex differences in K-COWS scores, suggesting that withdrawal severity is comparable between men and women when assessed systematically. This aligns with prior analyses indicating minimal sex effects on COWS totals once measurement invariance is considered (24). Although our study was not powered to detect small sex-related nuances, the absence of large differences supports the use of the same interpretive thresholds across sexes pending a larger confirmatory study.

There are several limitations in this study. First, the sample size was modest and drawn exclusively from tertiary pain-clinic settings among patients with prescription OUD, limiting generalizability to people using illicit opioids. Second, withdrawal severity clustered in the mild–moderate range, leaving performance at extreme severities less certain. Third, EFA and CFA were conducted in the same dataset, and the CFA fit shortfalls counsel caution about over-interpreting the two-factor solution. Another important limitation is that inter-rater reliability could not be estimated, because only a single clinician’s rating was available per patient. Future studies should include ratings from multiple independent clinicians to quantify inter-rater reliability for the K-COWS. Furthermore, test–retest reliability was not assessed; therefore, the temporal stability of the scale remains unknown. Importantly, the Korean context makes recruitment of illicit-opioid users challenging, which likely restricts symptom diversity. Addressing this will require multi-site collaborations and broader inclusion. Priorities for future research include (i) independent-sample CFA and Item Response Theory (IRT) analysis to verify the dimensional structure; (ii) assessment of measurement invariance by sex and age; (iii) determination of clinically actionable thresholds; and (iv) formal evaluations of inter-rater and test–retest reliability.

In conclusion, the K-COWS demonstrated strong reliability, good convergent and discriminant validity, and a plausible though not yet definitively confirmed two-factor structure for opioid withdrawal assessment in Korean clinical settings. Given the preliminary nature of the dimensional structure, the K-COWS total score remains the most practical and evidence-based measure for standardized withdrawal assessment. This tool can support clinical decisions and help align Korean opioid withdrawal management with international best practices.

## Data Availability

The raw data supporting the conclusions of this article will be made available by the authors, without undue reservation.
